# Anomaly Detection and Remaining Useful Life Estimation for the Health and Usage Monitoring Systems 2023 Data Challenge

**DOI:** 10.3390/s24134258

**Published:** 2024-06-30

**Authors:** Omri Matania, Eric Bechhoefer, David Blunt, Wenyi Wang, Jacob Bortman

**Affiliations:** 1 BGU-PHM Laboratory, Department of Mechanical Engineering, Ben-Gurion University of the Negev, P.O. Box 653, Beer Sheva 8410501, Israel; jacbort@bgu.ac.il; 2 GPMS International Inc., 93 Pilgram Place, Waterbury, VT 05676, USA; eric@gpms-vt.com; 3 Defence Science and Technology Group (DSTG), Department of Defence, Melbourne, VIC 3207, Australia; david.blunt@defence.gov.au (D.B.); wenyi.wang@defence.gov.au (W.W.)

**Keywords:** data-driven, deep learning, gear, vibration signals, digital twin

## Abstract

Gear fault detection and remaining useful life estimation are important tasks for monitoring the health of rotating machinery. In this study, a new benchmark for endurance gear vibration signals is presented and made publicly available. The new dataset was used in the HUMS 2023 conference data challenge to test anomaly detection algorithms. A survey of the suggested techniques is provided, demonstrating that traditional signal processing techniques interestingly outperform deep learning algorithms in this case. Of the 11 participating groups, only those that used traditional approaches achieved good results on most of the channels. Additionally, we introduce a signal processing anomaly detection algorithm and meticulously compare it to a standard deep learning anomaly detection algorithm using data from the HUMS 2023 challenge and simulated signals. The signal processing algorithm surpasses the deep learning algorithm on all tested channels and also on simulated data where there is an abundance of training data. Finally, we present a new digital twin that enables the estimation of the remaining useful life of the tested gear from the HUMS 2023 challenge.

## 1. Introduction

Gears are crucial components of rotating machinery that are expected to withstand long working periods [1,2]. The diagnosis of gears using vibration analysis involves four stages: fault detection, fault type classification, fault severity estimation, and the estimation of remaining useful life (RUL) [3,4], with many well-established methods [5,6] including processing stages such as angular resampling [7,8], transfer function estimation [9,10], synchronous average [11,12], condition indicators analysis [13,14], and models [15,16]. The current study consists of three parts: (1) the introduction of a new benchmark, (2) a comparison of traditional and data-driven algorithms of fault detection, and (3) the presentation of a new digital twin for RUL estimation.

In the first part of this study, in Section 2, a new endurance gear benchmark dataset is presented. This benchmark enables the examination of anomaly detection algorithms [17,18] and the severity and remaining useful life of estimation algorithms. The dataset was previously used in the HUMS 2023 conference data challenge [19]; in the current study, it was made publicly available, accompanied by a meticulous description for future research.

Fault detection has been extensively studied for various types of components throughout the years [7,20]. Two primary disciplines are commonly employed for fault detection: (1) traditional signal processing approaches [21,22,23] and (2) data-driven approaches [24,25]. Traditional approaches involve the utilization of signal processing algorithms to extract features representative of fault (condition indicators) [13,14], followed by the application of statistical or simple threshold methods. Data-driven approaches can be divided into two subcategories: deep learning [26,27] and classical machine learning [28]. In both subcategories, the focus is on learning directly from the data without relying on features defined by humans [26].

In contrast to the remaining stages of diagnosis for rotating components (i.e., fault classification [29,30], severity estimation [31], and RUL estimation [32,33]) that require faulty data (labeled or unlabeled) during the training phase [14], data-driven approaches for fault detection can be trained solely on healthy data. Additionally, it can be assumed that a sufficient amount of healthy data is available—which is crucial for the performance of data-driven approaches—due to the prolonged operation of rotating components in the healthy state. However, an interesting finding from the HUMS 2023 data challenge is that data-driven approaches do not surpass signal processing algorithms and, in fact, perform worse compared to cutting-edge traditional approaches. These results are presented and discussed in the second part of this paper (Section 3). The data challenge survey is followed by a quantitative comparison between a traditional signal processing algorithm and a standard, well-known, and well-performing deep learning architecture that result in the same conclusion (Section 4).

The last part of this paper, as described in Section 5, focuses on the RUL estimation of the HUMS 2023 benchmark dataset using a new digital twin. Digital twins are an emerging concept that find applications in a number of fields [34,35]. In this study, the aim is to demonstrate how this concept can address the curse of dimensionality problems associated with complex tasks like RUL estimation. Specifically, a new digital twin is employed, utilizing a signal processing algorithm to extract meaningful and updated health indicators of the rotating component, while a crack propagation model and statistical analysis are used for estimating the RUL of the system.

## 2. New HUMS 2023 Benchmark Dataset

**Figure 1:**
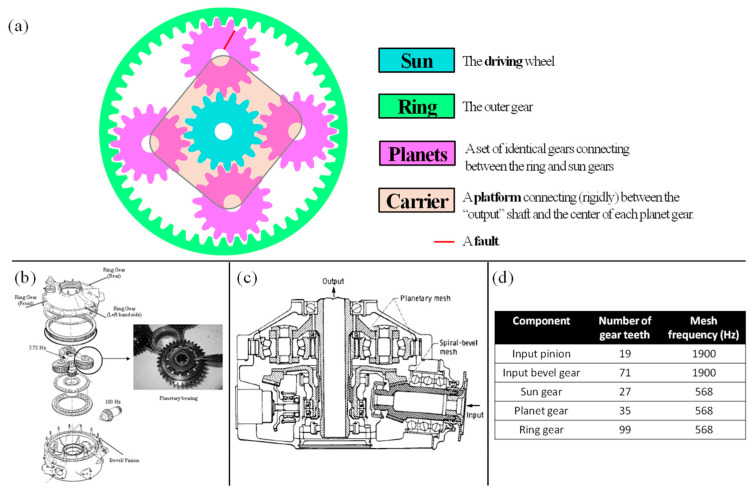
(**a**) An illustration of the planetary gear with the faulted planet gear (**b**) main rotor gearbox (three-planet version) of Bell 206B-1 (OH-58). The four-planet version was used in the test, but the configuration is essentially the same for the four-planet gearbox apart from differences in detail in the planet carrier, planet gears, and bearings. (**c**) The location of the planetary gearbox inside the main rotor. Reproduced from Ref. [40]. (**d**) Number of teeth and mesh frequencies of the gears.

The HUMS 2023 benchmark dataset was created with the purpose of investigating fatigue cracking in thin-rim helicopter planet gears (Figure 1a). These gears have a design where the gear body includes the outer raceway of the planet bearing, and the crack starts either at the raceway surface or in its immediate vicinity and then progresses through the gear body. Detecting this specific type of crack reliably poses a significant challenge, and it has the potential to result in the catastrophic failure of the main rotor gearbox (Figure 1b). Two helicopter accidents, namely the AS-332L2 Super Puma in 2009 [36] and the H-225 Super Puma in 2016 [37], were attributed to similar fault patterns.

Under controlled conditions, the seeded-fault test was conducted at the helicopter transmission test facility in Australia’s Defence Science and Technology Group (DSTG) [38]. The benchmark dataset was generated from a propagating fatigue crack in a planet gear within a helicopter’s main rotor gearbox. The specific tested model was the four-planet version of a Bell Kiowa 206B-1 (OH-58) [39] main rotor gearbox. This gearbox features two speed reduction stages: a spiral pinion/bevel gear stage and a planetary stage. The test itself operated at a nominal input speed of 6000 RPM, resulting in an output speed of 344 RPM. Detailed information about the gears and their mesh frequencies can be found in Figure 1c.

**Figure 2:**
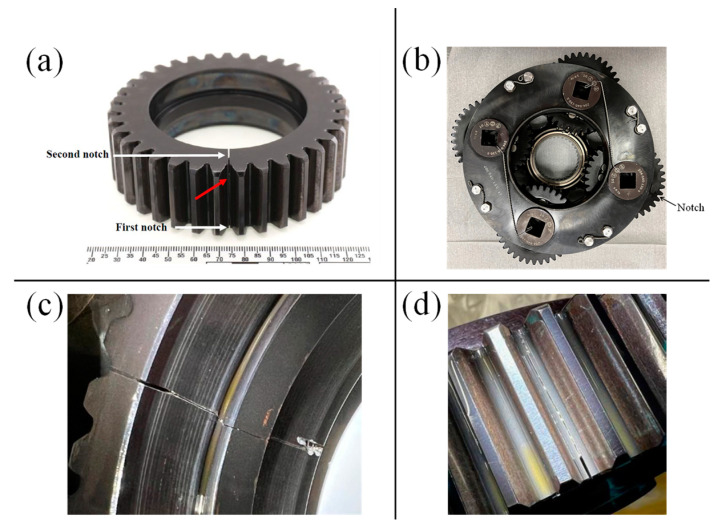
The gear fault. (**a**) The location of the notches and the propagation path (marked in red arrow), (**b**) the location of the gear and the notch, (**c**) a side picture of the notch, (**d**) the propagation path of the fault.

**Figure 3:**
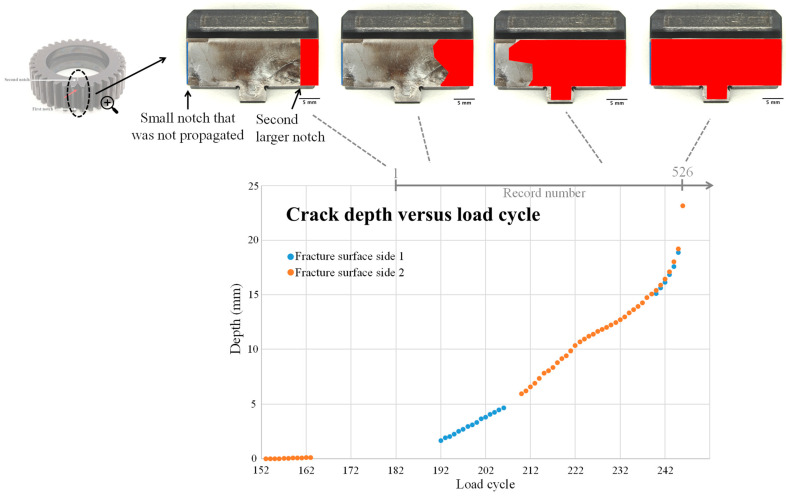
Illustration of the fault propagation throughout the experiment.

The cracked planet gear, depicted in Figure 2, exhibits two notches, one on each side. Initially, the first (smaller) notch did not result in crack initiation during the first 146 load cycles. Consequently, the gearbox was disassembled, and a second (larger) notch was introduced on the opposite side. This alteration successfully led to the initiation and propagation of a fatigue crack from the second notch, spanning load cycles 147 to 241. Figure 3 illustrates the fault propagation throughout the experiment.

During the experiment, the gear experienced periodic loads over a 30-min interval, comprising 2 min of 50% load, followed by 2 min of 75% load, 2 min of 100% load, and finally 24 min of 125% load (100% load = 303 Nm) at an input pinion shaft speed of 6000 RPM. The majority of the load cycle was spent in an overloaded condition to accelerate the propagation of the crack.

Following the conclusion of the experiment, the gear underwent fractography analysis to assess the fault progression over time. Two significant stages were identified by DSTG’s analysis: (1) the transition from initiation to consistent growth, observed in record #242, and (2) an accelerated crack growth, identified at record #457.

**Figure 4:**
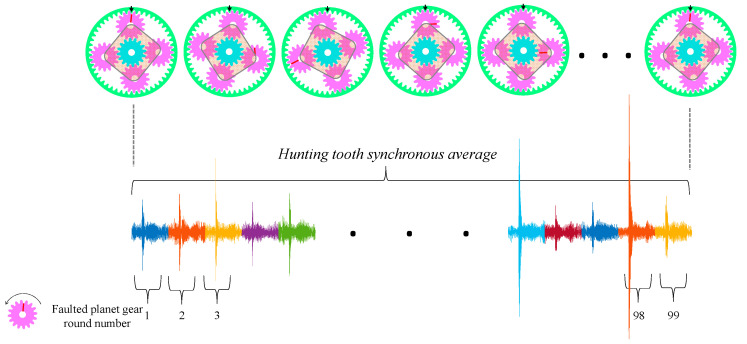
Illustration of hunting tooth synchronous average.

The HUMS 2023 benchmark dataset consists of a total of four sets of 526 hunting tooth synchronous averages (Figure 4) for each record over the span of seven days. Each of the four vibration channels is assigned 526 records. The data records were taken at three minutes interval within a manually controlled 30-min load cycle, all of them under a 125% load (i.e., 378.75 Nm). There is a gap of more than 10 min in the timestamp between data records from consecutive load cycles. These records represent the vibration signals of the gear during the last 60 load cycles (i.e., from load cycle #182 to load cycle #241), where each cycle produced between eight and nine records. Each record is obtained after angular resampling and hunting tooth synchronous averaging.

**Figure 5:**
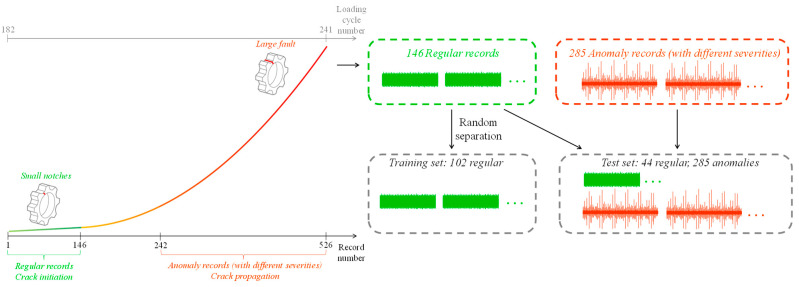
Separation of HUMS 2023 benchmark into training and test sets.

To examine anomaly detection algorithms, it is recommended to divide the benchmark into two categories based on the fractography analysis: regular data from 1 to 146 of crack initiation, and anomaly data from 242 to 526 of crack propagation as presented in Figure 5. This separation helps significantly reduce the error in severity estimation and allows for the assumption that the anomaly dataset represents a significantly different health condition. One advantage of this dataset is the availability of crack propagation estimation, thus the test–training separation strongly relates to the real health of the rotating component.

## 3. Survey of HUMS 2023 Challenge

A total of 14 groups participated in the HUMS 2023 data challenge [19], of which 11 groups were granted permission to upload their summary files. The approaches employed by these groups encompassed both traditional methods—which involved signal processing and analysis of condition indicators—and data-driven techniques, including deep learning and classical machine learning. Specifically, out of the 11 groups, seven utilized traditional approaches, while four groups employed data-driven methods (two groups utilized deep learning, and two groups utilized classical machine learning).

The survey of the suggested approaches was conducted by analyzing the reported results. The comparison process primarily relies on qualitative assessment based on these reported results, as the codes for most approaches are not available, and variations in the examination and training–test splits exist among different groups. Additionally, no analysis of the first anomaly detections is considered since it heavily relies on the designated probability of false alarm, which is not defined in most of the groups. Furthermore, it is influenced by the sizes of the training and test splits.

**Figure 6:**
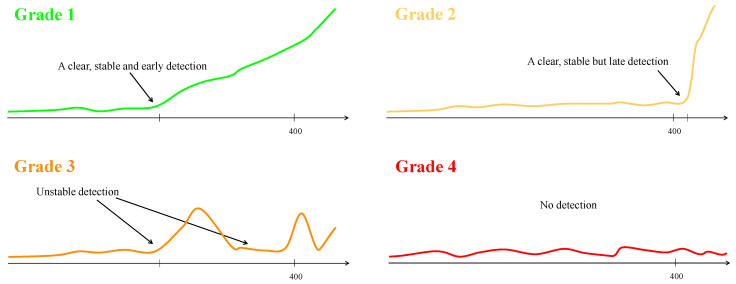
The four optional grades: (**1**) a clear, stable, and early detection; (**2**) a clear and stable detection but at a very late stage; (**3**) unstable detection; and (**4**) no detection.

The trends of the four channels were analyzed for each group, and the algorithm’s performance was categorized into four grades: Grade 1—clear detection of the fault before a late stage (record number 400). Grade 2—clear detection, but at a later stage (record number 400 or later). Grade 3—detection is not clear. Grade 4—No detection. Figure 6 illustrates these categories. For groups that analyzed all the channels together instead of separately, the grading was based on the combined performance of all channels. However, this is considered a less ideal scenario since a consistent performance across all four channels would be more convincing.

**Figure 7:**
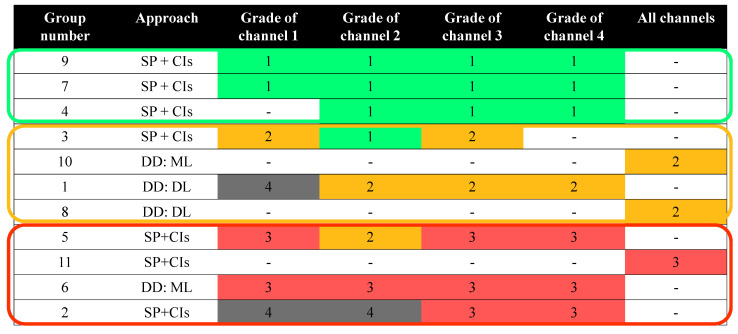
Separation of the 11 participating groups that granted permission to upload their summary files of the HUMS 2023 data challenge based on their performance into three categories: (1) groups with good results on most of the channels (green); (2) groups with mediocre results (orange); and groups with poor results (red). The grades of 1–4 for each channel are explained in Figure 6.

The results of the groups are summarized in Figure 7. They have been categorized into three categories: (1) groups with good results on most of the channels (green); (2) groups with mediocre results (orange); and groups with poor results (red). The ranking within each category is not significant as the differences between the groups are small. This is because the analysis based on the groups’ reports is not sensitive enough to differentiate between the results within the categories.

As depicted in Figure 7, none of the data-driven approaches were placed in the first category. Additionally, the performance of the data-driven approaches did not provide a clear, stable, and early detection of the fault. This finding is particularly significant for three reasons: the inherent advantages of traditional algorithms over data-driven approaches, the performance of data-driven approaches in other data challenges in various other fields, and the necessity for careful comparisons between data-driven approaches and traditional approaches when new data-driven methods are proposed.

Traditional approaches have a natural advantage over data-driven approaches, regardless of differences in performance. Traditional approaches possess several benefits: they can be easily explained, and can be applied across various operating conditions and applied across new cases. For instance, in many traditional algorithms, variations in parameters such as speed can be addressed by analyzing new frequencies of interest that can be computed without requiring new examples (as observed in bearing diagnosis, for instance [20,41]). Additionally, many traditional algorithms allow for a straightforward definition of a probability false alarm rate with well-defined boundaries, which is crucial for real-world applications where the trade-off between early detection and false alarms is significant. Furthermore, traditional approaches are inherently explainable compared to data-driven approaches. This is a critical aspect to consider, especially when it comes to translating the recommendations of these approaches into maintenance actions taken by humans. Some of these actions may be crucial in mitigating high risks, such as catastrophic failures that can potentially endanger human lives.

The results of the HUMS 2023 data challenge are also interesting when considering that in many fields where data-driven approaches are considered cutting-edge technologies, they outperform all other approaches by a significant margin in most data challenges. For instance, in image processing or natural language processing tasks, data-driven approaches consistently exhibit superior performance compared to other methods, and it is uncommon to find alternative approaches that surpass the performance of data-driven approaches in such challenges. For example, since 2012, with the rise of deep learning architectures for image classification, all the best-performing algorithms in the famous ImageNet challenge have been deep learning architectures [42,43,44,45]. This raises the question of whether data-driven approaches are indeed the leading technologies for fault detection tasks in the diagnosis of rotating machinery. If that were the case, it is surprising that not a single data-driven approach managed to secure a top ranking in Figure 7.

The two aforementioned paragraphs raise the question of whether new data-driven approaches are adequately compared to traditional approaches in research articles. It appears that, in many cases, including in other tasks and components such as bearing fault type diagnosis, data-driven approaches are solely compared amongst themselves and not against traditional approaches [46,47,48,49,50,51], despite the latter’s natural advantages and established performance. For instance, bearing fault detection is a well-known task that can be effectively addressed using traditional approaches, which have been implemented in real systems for over a decade. In the authors’ opinion, new data-driven approaches should be compared to traditional approaches as well, and not solely against other data-driven methods. Without demonstrating a superior performance over traditional approaches, there seems to be little justification for adopting a new data-driven approach, considering the inherent advantages of traditional methods.

## 4. A Quantitative Comparison between Traditional and DL Approaches

In this section, a quantitative comparison is made between a traditional fault detection algorithm, which consists of signal processing and condition indicator analysis, and a deep learning algorithm. First, the traditional algorithm is presented, followed by the deep learning approach. Subsequently, they are compared using the ROC-AUC metric [52] on both the HUMS 2023 benchmark dataset and a simulated dataset.

### 4.1. Traditional Fault Detection Algorithm

**Figure 8:**
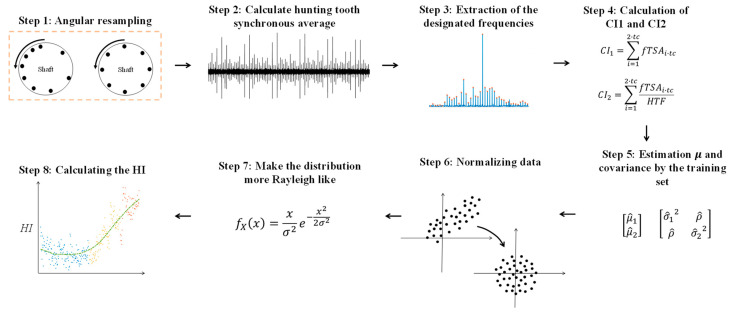
The traditional fault detection algorithm consists of signal processing and condition indicator analysis.

The traditional fault detection algorithm is based on signal processing and condition indicators analysis as depicted in Figure 8. The algorithm consists of the following steps:

1. The signal is angular resampled [7,53].
2. The synchronous average of the hunting tooth is calculated [11,12].
3. The designated frequencies of the gear mesh are extracted from the order.
4. Two condition indicators are extracted: $CI_{1}=\sum_{i=1}^{2\cdot tc}fTSA_{i\cdot tc}$ and $CI_{2}=\sum_{i=1}^{2\cdot tc}\frac{fTSA_{i\cdot tc}}{HTF}$, where tc is the hunting tooth count and $fTSA_{i\cdot tc}$ is the value of the hunting tooth synchronous average at frequency i·tc, and HTF is the value of the hunting tooth frequency.
5. The expectation and covariance of the extracted condition indicators are estimated based on the training set.
6. The data are normalized according to the estimated expectation and covariance of the former step.
7. The distribution of the condition indicators is made more Rayleigh-like [54,55]. That means that the empirical cumulative distribution function is made more Rayleigh-like by setting to zero the first five percent examples near the zero value.
8. The health indicator (HI) is calculated by the magnitude of the processed features, i.e., $HI_{n}=\sqrt{{\tilde{CI_{1}\;}}^{2}+{\tilde{CI_{2}\;}}^{2}}$, where $\tilde{CI_{1}}$ and $\tilde{CI_{2}}$ are the processed condition indicators after Steps 6 and 7.

### 4.2. The Deep Learning Approach

The deep learning approach is based on signal compression using an autoencoder (AE), which is commonly used in various tasks and has been shown to yield good results [26,56]. The training examples of healthy signals are divided into consecutive segments, which are then compressed using an encoder and decompressed by a decoder. The AE is trained to minimize the mean squared error (MSE) [28,57,58] between the segments before and after compression, and the HI is calculated based on the mean squared error between the original signal and the reconstructed signal consisting of consecutive segments.

To increase the sample complexity for improving generalization abilities, the records were divided into segments corresponding to each round of the faulted gear. This was done to address the low sample complexity issue present in the HUMS 2023 benchmark. For the HUMS 2023 benchmark, each record was divided into 99 segments, and for the simulated dataset, it was divided into 17 segments. The architecture of the AE can be found in Ref. [59], where the optimizer was Adam, and the training was halted when the error on the validation set was not improved for at least three complete epochs.

**Figure 9:**
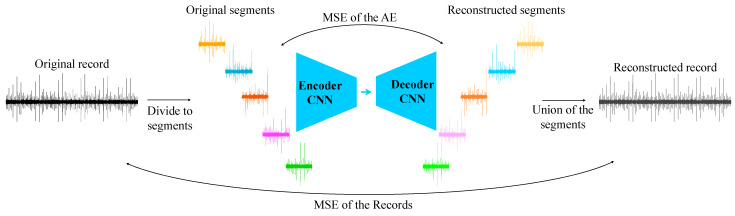
Illustration of the suggested algorithm for fault detection based on an AE. The records are separated to N segments (99 for HUMS 2023 benchmark and 17 for the simulated dataset) and the AE is trained to reconstruct the segments with minimum MSE error. Then, the MSE between the original record and the reconstructed record is calculated and defined as the HI.

The AE was trained on all the segments of the records in the training set. For example, if there are 1000 records in the training set and each record is divided into 17 segments, the training set size for the AE would be 17,000. The algorithm is illustrated in Figure 9. A higher MSE between the tested record and the reconstruction error indicates a fault because the AE is unable to effectively reconstruct new examples that deviate from the original healthy distribution.

### 4.3. Result on HUMS 2023 Benchmark Dataset

The HUMS 2023 benchmark dataset was used to compare the traditional and deep learning approaches quantitatively. The training and test sets were generated according to the depiction in Figure 5. The performance of each algorithm was evaluated using ROC-AUC [52], which is a common metric for deep learning-based anomaly detection algorithms.

**Figure 10:**
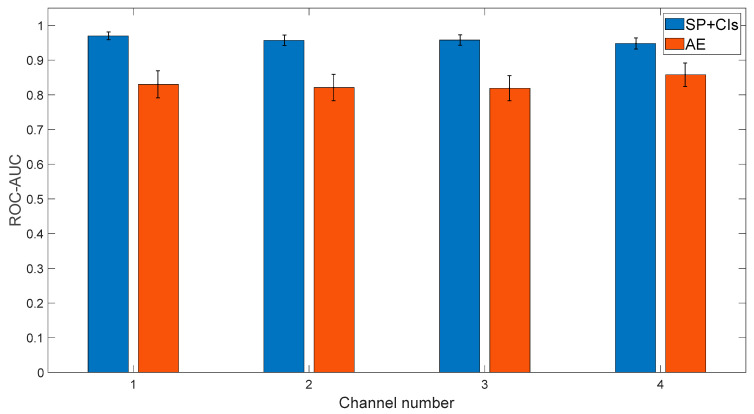
Result of the deep learning approach based on the traditional algorithm based on signal processing (SP) and condition indicators (CIs) analysis and the AE.

Each algorithm was tested on all four channels, with 100 repetitions of randomly splitting the healthy examples into training and test sets (the indices of the random separation can be found in Ref. [59]). The results are presented in Figure 10. As observed from the figure, in all cases, the traditional algorithm outperforms the standard, well-known deep learning approach that typically yields good results. This quantitative examination finding reinforces the conclusion of Section 3, which states that currently, deep learning approaches do not surpass traditional algorithms.

### 4.4. Result on Simulated Dataset

Deep learning algorithms require a sufficient amount of data to effectively generalize to new cases. This is due to the well-known tradeoff between bias and complexity, as their hypothesis class possesses a high VC dimension owing to their expressive power. In this study, it was confirmed that increasing the amount of data does not enable the deep learning approach to surpass the traditional algorithm. Therefore, a simulated dataset was generated where data could be generated without limitations.

**Figure 11:**
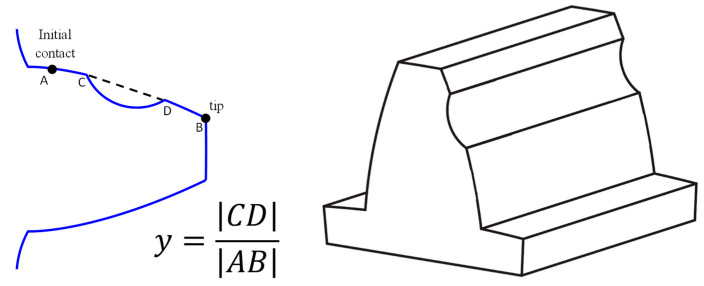
An illustration of the simulated pitting fault. (**Left**)—quantitative definition of the fault size as the ratio between the fault length $\left|CD\right|$ and the contact length $\left|AB\right|$. (**Right**)—an isometric view of the pitted tooth. Reproduced from Ref. [14].

The dataset consisted of records of both healthy signals and signals with a type of pitting fault, ranging in size from 0.034 to 0.378. The fault size is defined in Figure 11. To challenge the fault detection, white noise was added to the signals. The simulated data were generated using a dynamic model presented in Ref. [60].

The performance of the traditional algorithm and the deep learning algorithm was compared using an increasing number of training examples. The test set consisted of 500 healthy examples and 500 faulty examples. The AE was trained for 200 epochs or until there were no improvements observed in the validation set for at least three complete epochs. In all cases, the AE did not reach the limitation of 200 epochs, indicating that it achieved its best performance on the validation set.

**Figure 12:**
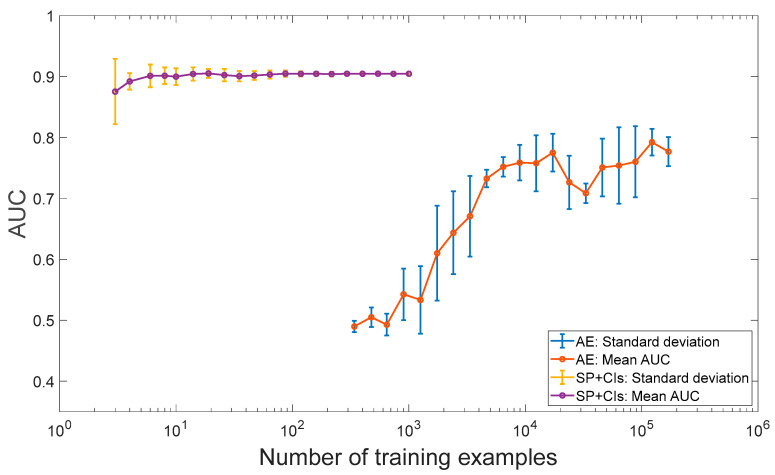
Results of the deep learning approach based on the AE and the traditional algorithm based on signal processing (SP) and condition indicators (CIs) analysis of the simulated data. The number of training examples of the AE is the number of segments in the training set. Each case was repeated 10 times with different healthy examples to examine the variance.

As depicted in Figure 12, the performance of the traditional algorithm quickly stabilizes and reaches an AUC slightly above 0.9 with only around 20 examples. On the other hand, the AE demonstrates an initial improvement, starting from an AUC of approximately 0.5 (which is equivalent to random guessing) and reaching an AUC near 0.75, as expected due to the increase in the number of examples. However, the AE’s performance remains significantly lower than that of the traditional algorithm. This example demonstrates that even with a large amount of data, the deep learning approach is still unable to surpass the performance of the traditional algorithm.

## 5. The New Digital Twin

A new digital twin has been developed for the RUL estimation of the planet gear rim crack in the HUMS 2023 benchmark dataset. This digital twin serves as a digital replica of the actual twin, calculating the health status of the gear for each record and utilizing the dislocation crack propagation theory model to estimate the RUL. Digital twins come in various forms and have numerous applications. In the current scenario, the digital twin is employed to tackle the complex problem of RUL estimation by overcoming the curse of dimensionality. The RUL estimation problem involves high dimensionality due to the abundance of former records with numerous potential features, which can be used to predict the RUL. The digital twin simplifies the problem by fusing all the previous cumulative data into a single parameter that represents the crack size. This parameter is then used to predict the RUL by simulating the propagation of the crack using the dislocation model.

**Figure 13:**
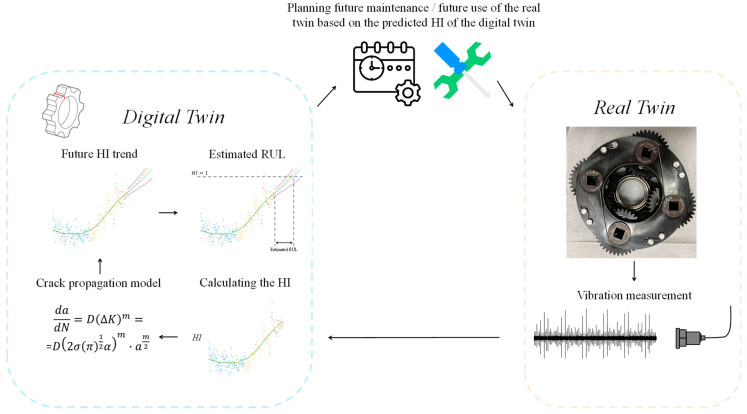
A block diagram of the real and digital twins: The measured data from the real twin are processed online by the digital twin. The digital twin generates predicted HI trends and estimates RUL, which are then utilized for making maintenance and usage decisions for the real twin.

The digital twin uses the measured vibration data to calculate a health indicator through signal processing algorithms that extract features related to spall severity and statistical calculations. Then, the digital twin employs a crack propagation model based on Paris’s law to estimate the current state of the fault and predict its progression. Using a prediction algorithm, the RUL is estimated. Figure 13 illustrates the DT process, which consists of the following steps:

1. The HI is calculated based on the measured signal of the real twin. It is assumed that the HI is correlated to the crack size.
2. A crack size propagation model is used to predict the future HI trend. The model assumes $\frac{da}{dN}=D{\left(\Delta K\right)}^{m}=D{\left(2\sigma {\left(\pi \right)}^{\frac{1}{2}}\alpha \right)}^{m}\cdot a^{\frac{m}{2}}$, where a is the crack size, N is the number of loading cycle, D is the material constant, m is the crack growth exponent, which is 4 for steel, σ is the delta strain, and α is a correction factor due to the shape of the component.
3. The RUL is estimated when the HI will reach the value of 1.

**Figure 14:**
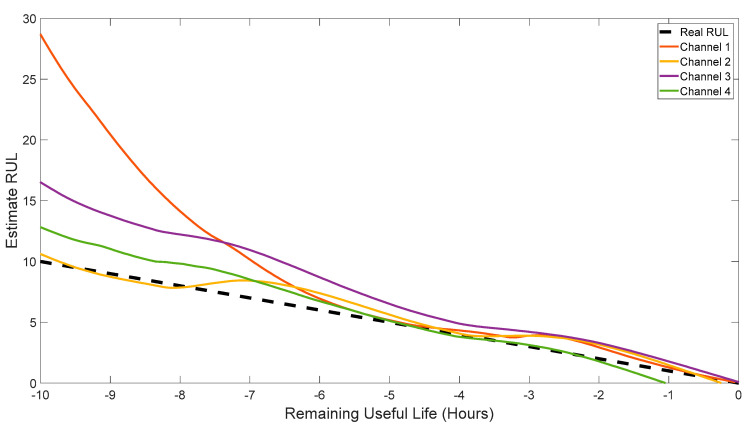
Estimated RUL of the digital twin based on each channel separately. I.e., the RUL was estimated for each channel based on the time it reaches HI of 1.

Figure 14 depicts the real RUL and the estimated RUL using the digital twin for all four channels. The dataset comprised 526 acquisitions for each channel, equivalent to 26.25 h of accelerated life testing (with a record taken every three minutes). Earlier in the run, there is little measured degradation, so that $\frac{da}{dN}$ is small and the RUL is large. As the fault propagates, the RUL decreases and approaches the $\frac{dRUL}{dt}$ of −1 (e.g., for each hour of life run, one hour of component life is consumed). The plot from 10 h until 0 h reflects the point where the digital twin’s estimate or RUL has converged and is providing good data. From a maintenance perspective, this allows operators/maintainers to marshal resources (order a gearbox, tools, the required skill set needed to perform the maintenance) and to schedule that maintenance. Unscheduled maintenance results in missing income, and for a helicopter, could potentially lead to a mishap. Another benefit of removing the gearbox from operations prior to failure is a lower cost of repair. From the figure, it is evident that the new digital twin is highly effective in estimating the RUL across all channels, and the accuracy of the estimation improves as the remaining time approaches 5 h and below. Note that this is an aggressive, accelerated test. In many real-world applications, with a much longer operational life, the digital twin may be able to provide an RUL prediction of 150 to 700 h.

## 6. Summary

In this article, a new dataset called the HUMS 2023 benchmark produced by DSTG was introduced and made available. The HUMS 2023 data challenge contest results were analyzed, leading to an interesting conclusion that data-driven approaches currently do not outperform the best-performing traditional techniques, which combine signal processing and condition indicators. This conclusion was reinforced by a quantitative comparison of a traditional algorithm and a well-known data-driven approach based on deep learning algorithms using the HUMS 2023 benchmark. Additionally, this conclusion was supported by a comparison on a simulated dataset where training examples were abundantly available.

A new digital twin was developed to address the challenge of estimating the RUL. The digital twin calculates the HI and utilizes crack propagation theory to predict the time at which the HI will reach the critical value of 1. The digital twin demonstrates a good performance across all four tested channels in the HUMS 2023 benchmark.

## Data Availability

The data presented in this study are available on request from the corresponding author.

## Abbreviations

AE	autoencoder
DSTG	Defence Science and Technology Group
HI	health indicator
MSE	mean squared error
RUL	remaining useful life
